# Fibrinogen-to-albumin ratio predicts mortality in patients with diabetes mellitus and atherosclerotic cardiovascular disease

**DOI:** 10.3389/fendo.2025.1539114

**Published:** 2025-05-29

**Authors:** Haixu Yu, Yijian Fan, Jihong Wang, Wei Liu

**Affiliations:** Department of Cardiology, Beijing Jishuitan Hospital, Capital Medical University, Beijing, China

**Keywords:** fibrinogen-to-albumin ratio, atherosclerotic cardiovascular disease, prognosis, mortality, diabetes mellitus

## Abstract

**Background:**

Circulating fibrinogen-to-albumin ratio (FAR) has been proposed as a novel inflammatory biomarker in patients with heart failure and malignant tumor. However, the prospective association in patients with atherosclerotic cardiovascular disease (ASCVD) remains to be investigated.

**Methods:**

The trial enrolled 821 patients with ASCVD from United States National Health and Nutrition Examination Survey from 1999-2002, with mortality follow-up through December 31st, 2019. Participants were divided into high-level FAR (FAR_H) and low-level FAR (FAR_L) groups. Kaplan-Meier and Cox proportional risk models were used to investigate the relationship between FAR and all-cause mortality in middle-aged and older persons with diabetes mellitus (DM).

**Results:**

During the median follow-up time of 15.6 years, 608 (weighted, 74.1%) ASCVD patients developed mortality. The incidence of mortality differed significantly between the FAR groups in patients with or without DM. Compared with FAR_L group, the FAR_H group has increased 90% mortality in the ASCVD patients with DM (HR = 1.90, 95%CI 1.22 - 3.00; P = 0.005). Moreover, the FAR and DM have a significant interaction (P = 0.016) in the ASCVD patients.

**Conclusions:**

In this prospective cohort study, a higher level of FAR was independently associated with 15-year mortality among ASCVD patients with DM. The combination of FAR and DM in ASCVD patients may aid in risk stratification and prognosis.

## Introduction

Despite significant improvements in management and prevention strategies in recent years, cardiovascular disease (CVD) remains the leading cause of death and disability worldwide, with two-thirds attributed to atherosclerotic cardiovascular disease (ASCVD) (1). According to a report from the World Health Organization, a total of 17.9 million people died from ASCVD in 2019, which accounting for 32% of all deaths in the world (2). Rising mortality in middle and late adulthood indicates ASCVD, which can result in a population-wide fall in life expectancy (3). As the highest number of CVD deaths in countries throughout the world, ASCVD in United States in challenged by the multiple pressures of population aging and the constant increase in the prevalence of cardiometabolic abnormalities, hypertension, hyperlipidemia, and diabetes mellitus due to lifestyle, and social risk factors including smoking et al. Therefore, it is crucial for cardiovascular patients to identify, target, and monitor at the population level (4).

Systemic vascular inflammation plays multiple maladaptively pathophysiological process in the progression and destabilization of atherosclerosis (5). As the chronic inflammatory condition caused by cholesterol, atherosclerotic progression can lead to the formation of arterial plaque, rupture, and thrombosis (6). In stable ASCVD, many patients appeared atheroprogression are attributed to elevated levels of vascular inflammatory markers (7). Inflammation can increase the risk of developing ASCVD, regardless of lipid profile. Anti-inflammatory drugs, such as colchicine, canakinumab, and methotrexate, have been shown to effectively reduce the risk of developing ASCVD (8, 9). Previous studies have suggested that inflammatory markers are reliable prognostic biomarkers in patients with cardiovascular disease and heart disease, as well as diabetes mellitus. The presence of insulin resistance is a hallmark of type 2 diabetes, leading not only to hyperglycemia but also to low-grade inflammation. The low-grade inflammatory state influences atherogenesis by accelerating foam cell formation and low-density lipoprotein cholesterol (LDL-C) uptake, ultimately further influencing the progression of cardiovascular disease (10). High fasting glucose and type 2 diabetes are recognized as major risk factors for ASCVD. Therefore, the combination of ASCVD with diabetes may further exacerbate cardiovascular damage and increase the likelihood of myocardial infarction and heart failure.

The current clinical assessment of inflammation includes white blood cell count and classification, erythrocyte sedimentation rate, and C-reactive protein (11, 12). Fibrinogen-to-albumin ratio (FAR), defined as the ratio of fibrinogen to albumin, is a novel inflammation indicator that reflects the degree of inflammation in an individual, and it is considered to be prognostic factor for vascular diseases (13). FAR and C-reactive protein to albumin ratio have emerged as prognostic immune biomarkers for a variety of diseases, such as solid tumors, leukemia, hypertension, heart failure, ST-segment elevation, myocardial infarction, and sepsis (14–17). Özdemir et al. showed that FAR was a more effective predictor than fibronectin and albumin in the diagnosis of the exaggerated morning blood pressure surge (14).

However, there has been little research conducted on the relationship between FAR and all-cause mortality in patients with ASCVD. Therefore, this study was designed to assess the prognostic value of FAR levels in patients with ASCVD and specifically analyze ASCVD patients with diabetes in a subgroup.

## Methods

### Study design and population

In this study, we used publicly available data from the 1999-2002 National Health and Nutrition Examination Survey (NHANES), a cross-sectional, complex, multistage survey developed and administered by the National Center for Health Statistics of the U.S. Centers for Disease Control and Prevention, which enables representative estimates of U.S. population-related data. The NHANES study was required to receive full review and ethical approval from the National Center for Health Statistics Research Ethics Evaluation Committee.

**Figure 1:**
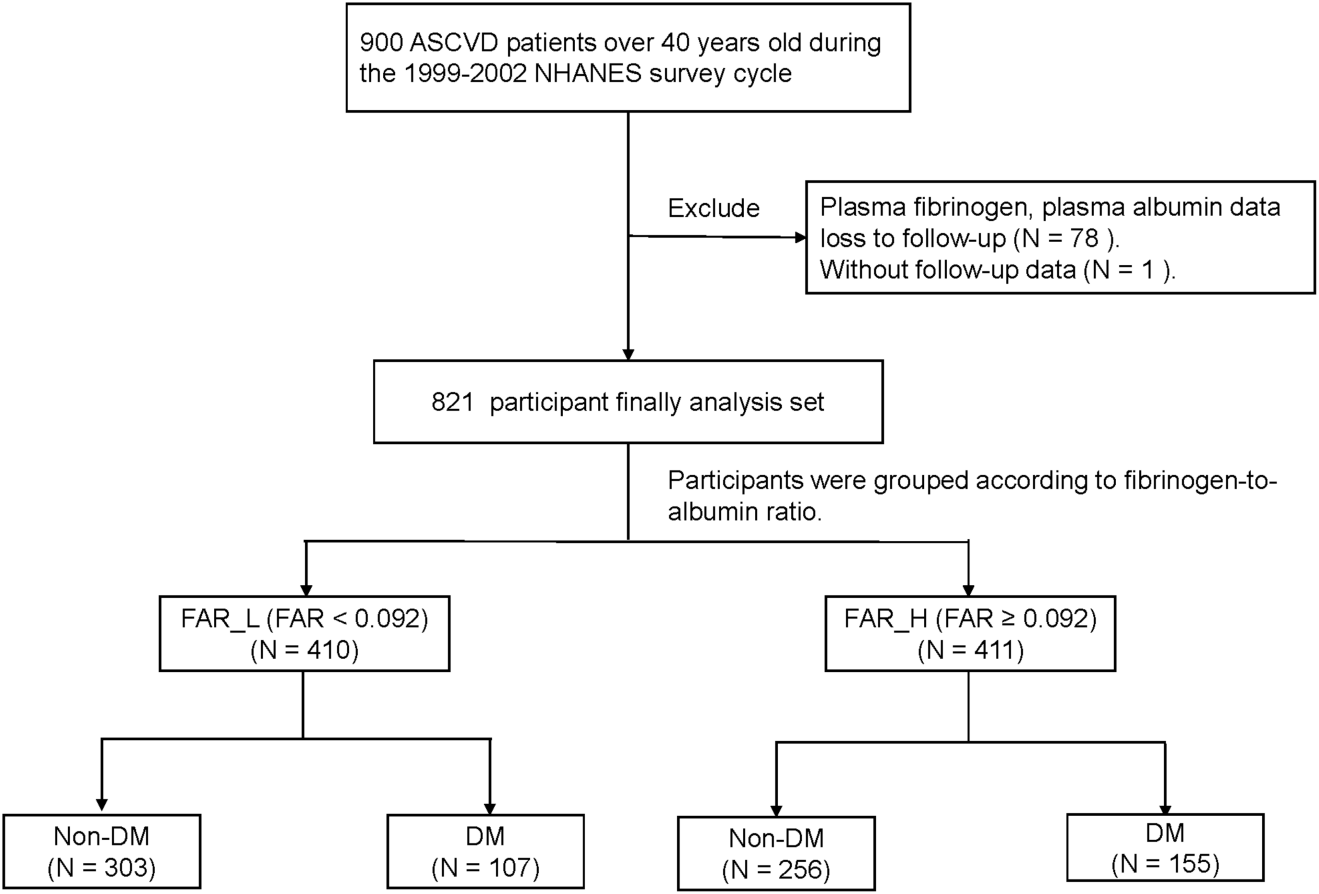
The flow chart of participant selection. FAR, fibrinogen-to-albumin ratio; ASCVD, arteriosclerotic cardiovascular disease; NHANES, National Health and Nutrition Examination Survey; DM, diabetes mellitus.

900 patients over 40 years old with ASCVD were enrolled in the NHANES study from 1999-2002 (**Figure 1**). 78 patients were excluded due to loss of plasma fibrinogen and plasma albumin data at the follow-up time, and 1 additional patient was lost to follow-up. As a result, 821 participants were finally included in this investigation with mortality follow-up until December 31, 2019.

### Exposure

FAR was derived from the baseline data, and it was calculated as the ratio of the plasma fibrinogen level (g/L) to the serum albumin level (g/L). Diabetes mellitus is categorised as type I diabetes, type II diabetes, secondary sexual diabetes and gestational diabetes. This study mainly included patients with type II diabetes mellitus. Diabetes was diagnosed using the diagnostic criteria listed below: (1) a hemoglobin A1c (HbA1c) level ≥ 6.5% (47.5 mmol/mol), or (2) fasting plasma glucose (FPG) level ≥ 7.0 mmol/L, or (3) random blood glucose ≥ 11.1 mmol/L, or (4) 2-h oral glucose tolerance test plasma glucose ≥ 11.1 mmol/L, or (5) the usage of hypoglycemic drugs, or (6) participants with a self-reported diabetes diagnosis. And ASCVD was defined as the presence of at least one diagnosis of coronary heart disease, angina, heart attack, or stroke. Based on the optimal critical value of FAR (FAR = 0.092) obtained from the restricted cubic spline function analysis, the study population was split into two groups: a high FAR group (FAR ≥ 0.092, FAR_H, N=411) and a low FAR group (FAR < 0.092, FAR_L, N=410). The FAR_H and FAR_L groups were further subgrouped based on whether or not they had diabetes mellitus and were split into four groups: (1) FAR_L/non-DM group; (2) FAR_L/DM group; (3) FAR_H/non-DM group; and (4) FAR_H/DM group.

### Defining variables of interest

The following details were self-disclosed: age, gender, ethnicity, education level, status as a smoker, alcohol use, DM, hypertension, CVD history, and family histories of CVD and DM. After the patients were fasted for more than nine hours, venous blood was drawn and analyzed in the laboratory to determine FPG concentrations. Triglyceride (TG) and total cholesterol (TC) concentrations were determined using enzymatic assays. When physical examination information was collected, participants’ high-density lipoprotein cholesterol (HDL-C) and low-density lipoprotein cholesterol (LDL-C) levels were collected as well as blood pressure. When participants had multiple blood pressure measurements the average of the multiple measurements was taken.

### Mortality outcomes

By looking up the ICD-10 numbers I00-I078 for each cause of death among study participants, a clear classification of all causes of death was achieved. All-cause mortality in patients with ASCVD was the primary outcome metric in our study. As of December 31, 2019, individuals in the NHANES between 1999 and 2002 had mortality follow-up data.

### Statistical analyses

Data processing was calculated after weighting according to the NHANES Analysis and Reporting Guidelines (https://wwwn.cdc.gov/nchs/nhanes/analyticguidelines.aspx#analytic-guidelines), taking into account complex multistage probability sampling. Continuous variables were assessed using analysis of variance and the means and standard errors of these variables are reported. Categorical variables were assessed using chi-square tests and percentages of categorical variables were reported.

Restricted cubic spline regression analysis was used to obtain the best FAR. Patient survival was assessed using standard Kaplan-Meier plots. Weighted univariate and multivariate Cox proportional risk models were used to estimate risk ratios (HR) and 95% confidence intervals (CI) for all-cause mortality in ASCVD patients. Model 1 was the base design and did not adjust for any confounders. The effects of age, sex, and race on outcomes were considered in Model 2. Model 3 builds on model 2 by considering the following confounders: education, smoking, alcohol consumption, BMI, familial cardiovascular disease, familial diabetes mellitus, hypertension, hyperlipidemia, and neoplasia. In this study, a subgroup analysis of patients with ASCVD combined with diabetes was also performed to assess the predictive significance of FAR for all-cause mortality in a population with or without ASCVD combined with diabetes.

A two-sided *P* value of 0.05 indicated statistical significance. All statistical analyses were performed in R (version 4.3.1, Vienna, Austria) software.

## Results

The fundamental characteristics of the two groups were characterized by dividing the individuals according to the ideal critical value of FAR (**Table 1**). Participants in the FAR_H group tended to be overweight (*P*-value < 0.001). And HbA1c, FPG, and DM were higher in the FAR_H group (all *P*-value < 0.001). Moreover, other baseline characteristics did not differ between the two groups, making them comparable. Participants were further categorized using FAR and whether or not they had diabetes (DM vs. nonDM). The **Supplementary Table S1** shows BMI, HbA1c, FPG, sex, education, family history of diabetes, and DM differed between the four groups.

**Table 1:** Baseline characteristics of study participants according to FAR.

Characteristics	Total	FAR_L	FAR_H	*P*-value
Age, years	66.132 (0.792)	64.102 (0.819)	68.580 (1.186)	0.001
BMI, kg/m^2^	29.301 (0.302)	28.326 (0.281)	30.535 (0.517)	< 0.001
SBP, mmHg	133.942 (1.123)	132.162 (1.423)	136.088 (1.392)	0.029
DBP, mmHg	69.922 (0.777)	70.808 (1.062)	68.854 (1.021)	0.168
HbA1c, %	5.957 (0.038)	5.816 (0.054)	6.128 (0.059)	< 0.001
fasting blood glucose, mmol/L	6.038 (0.116)	5.827 (0.143)	6.293 (0.171)	0.032
low-density lipoprotein cholesterol, mmol/L	3.041 (0.077)	2.942 (0.075)	3.183 (0.125)	0.067
triglyceride, mmol/L	1.988 (0.078)	2.083 (0.114)	1.871 (0.088)	0.142
Gender				< 0.001
Female, n (%)	337 (41.048)	139 (39.187)	198 (54.411)	
Male, n (%)	484 (58.952)	271 (60.813)	213 (45.589)	
Race				0.005
Black, n (%)	143 (17.418)	45 (5.414)	98 (13.585)	
Mexican American, n (%)	134 (16.322)	72 (2.694)	62 (2.973)	
Other, n (%)	43 (5.238)	21 (5.871)	22 (7.128)	
White, n (%)	501 (61.023)	272 (86.021)	229 (76.315)	
Alcohol.user, n (%)				0.017
0	128 (16.410)	50 (13.699)	78 (19.349)	
1	652 (83.590)	343 (86.301)	309 (80.651)	
Hypertension, n (%)				0.767
0	60 (7.308)	31 (9.416)	29 (8.504)	
1	761 (92.692)	379 (90.584)	382 (91.496)	
Edu, n (%)				0.040
less than 12 years	352 (42.979)	163 (30.060)	189 (40.278)	
12 years	286 (34.921)	144 (42.797)	142 (39.903)	
more than 12 years	181 (22.100)	103 (27.143)	78 (19.820)	
FamilyCVD, n (%)				0.864
0	675 (82.217)	336 (78.422)	339 (79.053)	
1	146 (17.783)	74 (21.578)	72 (20.947)	
FamilyDM, n (%)				0.396
0	405 (49.330)	206 (50.016)	199 (46.515)	
1	416 (50.670)	204 (49.984)	212 (53.485)	
DM, n (%)				< 0.001
0	559 (68.088)	303 (78.661)	256 (67.854)	
1	262 (31.912)	107 (21.339)	155 (32.146)	
Smoke, n (%)				0.361
non-smoking	330 (40.195)	155 (36.309)	175 (42.187)	
previous smoking	361 (43.971)	193 (44.127)	168 (38.680)	
smoking now	130 (15.834)	62 (19.565)	68 (19.133)	
Hyperlipidemia, n (%)				0.401
0	106 (12.911)	53 (9.940)	53 (12.163)	
1	715 (87.089)	357 (90.060)	358 (87.837)	
Cancer, n (%)				0.548
0	664 (80.877)	334 (81.928)	330 (80.035)	
1	157 (19.123)	76 (18.072)	81 (19.965)	

**Table 1** Baseline characteristics of study participants according to FAR (cont).

FAR, fibrinogen-to-albumin ratio; BMI, body mass index; SBP, systolic blood pressure; DBP, diastolic blood pressure; HbA1c, glycated hemoglobin; CVD, cardiovascular disease; DM, diabetes mellitus.

**Figure 2:**
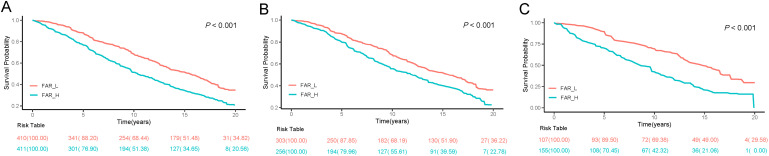
Kaplan–Meier analysis for all-cause mortality according to FAR in all people **(A)**, non-DM people **(B)** and DM people **(C)**. FAR, fibrinogen-to-albumin ratio; DM, diabetes mellitus.

**Figure 3:**
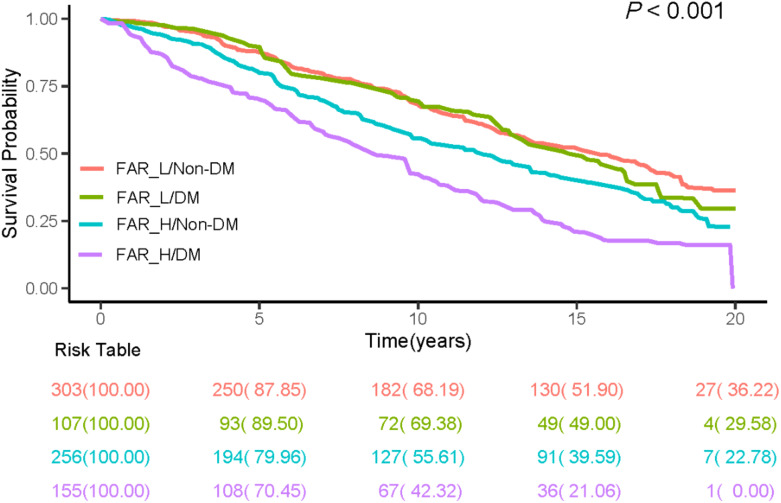
Kaplan–Meier analysis for all-cause mortality according to FAR and DM in all people. FAR, fibrinogen-to-albumin ratio; DM, diabetes mellitus.

There was a strong association between FAR and all-cause mortality among participants. Based on the Kaplan-Meier survival analysis, There was a significant difference between FAR groups in terms of all-cause mortality for all patients and patients with or without diabetes. (*P*-value < 0.001; **Figure 2**). Elevated FAR levels increased the risk of all-cause mortality by 55% in total ASCVD patients (HR = 1.55, 95%CI 1.14 - 2.10; *P*-value = 0.005) and 91% in ASCVD patients with DM (HR = 1.91, 95%CI 1.21 - 3.02; *P*-value = 0.010). Elevated FAR levels increase the risk of all-cause mortality by 90% in ASCVD diabetics (HR = 1.90, 95%CI 1.22 - 3.00; *P*-value = 0.005) after adjusting for all covariates. Additionally, all-cause mortality was not significantly higher in the FAR_H group compared to the FAR_L group in the no-DM population (HR = 1.01, 95%CI 0.73 - 1.39; *P*-value = 0.960) and overall population (HR = 1.15, 95%CI 0.87 - 1.52; *P*-value = 0.340). FAR and mortality were not correlated, whether or not the moderating variable was in the no-DM group. Furthermore, there was a significant interaction between FAR and DM in patients with ASCVD (*P* = 0.016; **Table 2**). Additionally, based on the results of analyses in the full ASCVD population according to FAR levels and DM, the Kaplan-Meier curves revealed that there was no significant difference in all-cause mortality for FAR_L/DM compared to FAR_L/Non-DM up to 15 years after diagnosis. The FAR_H/DM group had a significantly lower survival rate than the others out of the four groups based on FAR and DM (**Figure 3**).

**Table 2:** Cox single and multifactor regression analyses for all-cause mortality according to FAR in all people, non-DM people and DM people.

Glucose regulation state	Model 1		Model 2		Model 3		*P*-interaction
	HR (95% CI)	*P*-value	HR (95% CI)	*P*-value	HR (95% CI)	*P*-value	
Total	1.55 (1.14,2.10)	0.005	1.27 (0.99,1.62)	0.060	1.15 (0.87,1.52)	0.340	0.016
No-DM	1.39 (0.99,1.97)	0.060	1.11 (0.84,1.47)	0.450	1.01 (0.73,1.39)	0.960	
DM	1.91 (1.21,3.02)	0.010	1.81 (1.24,2.66)	0.002	1.90 (1.22,3.00)	0.005	

Model 1: Unadjusted; Model 2: Adjusted for age, gender, and race; Model 3: Adjusted for age, gender, race, education, smoking, alcohol consumption, BMI, familial cardiovascular disease, familial diabetes, hypertension, hyperlipidaemia, cancer. FAR, fibrinogen-to-albumin ratio; DM, diabetes mellitus; HR, hazard ratio; CI, confidence interval; P-interaction, interaction between FAR and DM.

**Figure 4:**
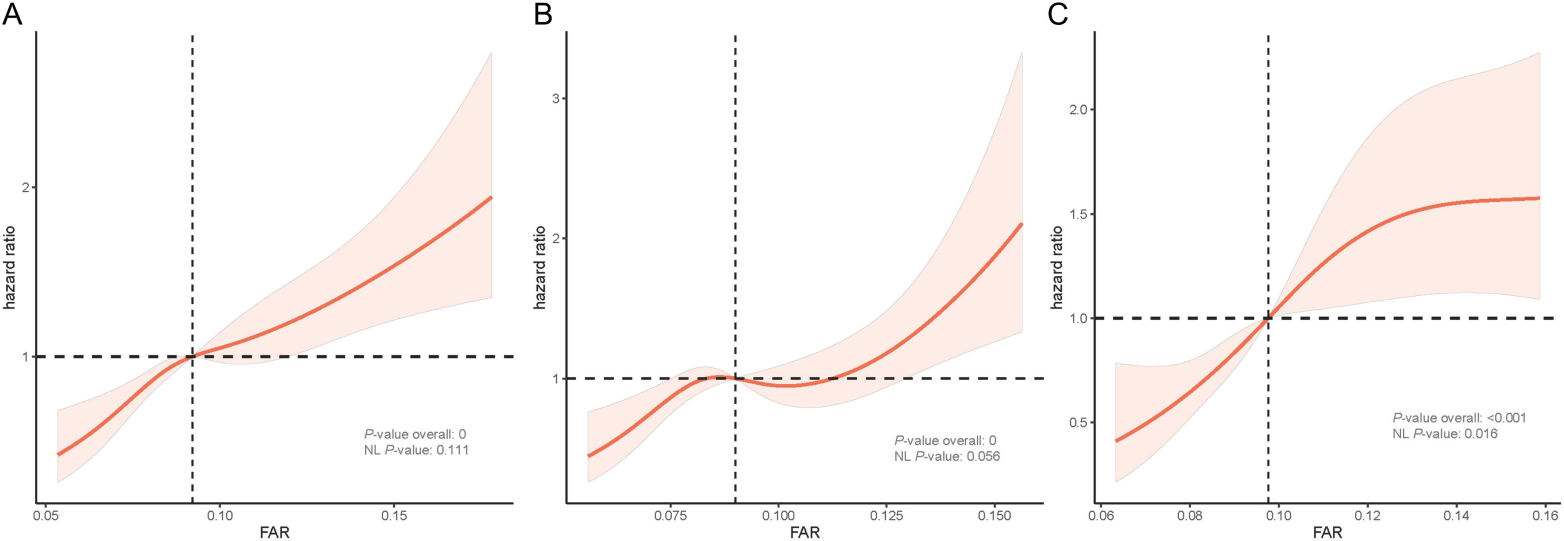
RCS results according to FAR in all people **(A)**, non-DM people **(B)** and DM people **(C)**. FAR, fibrinogen-to-albumin ratio; DM, diabetes mellitus.

**Figure 5:**
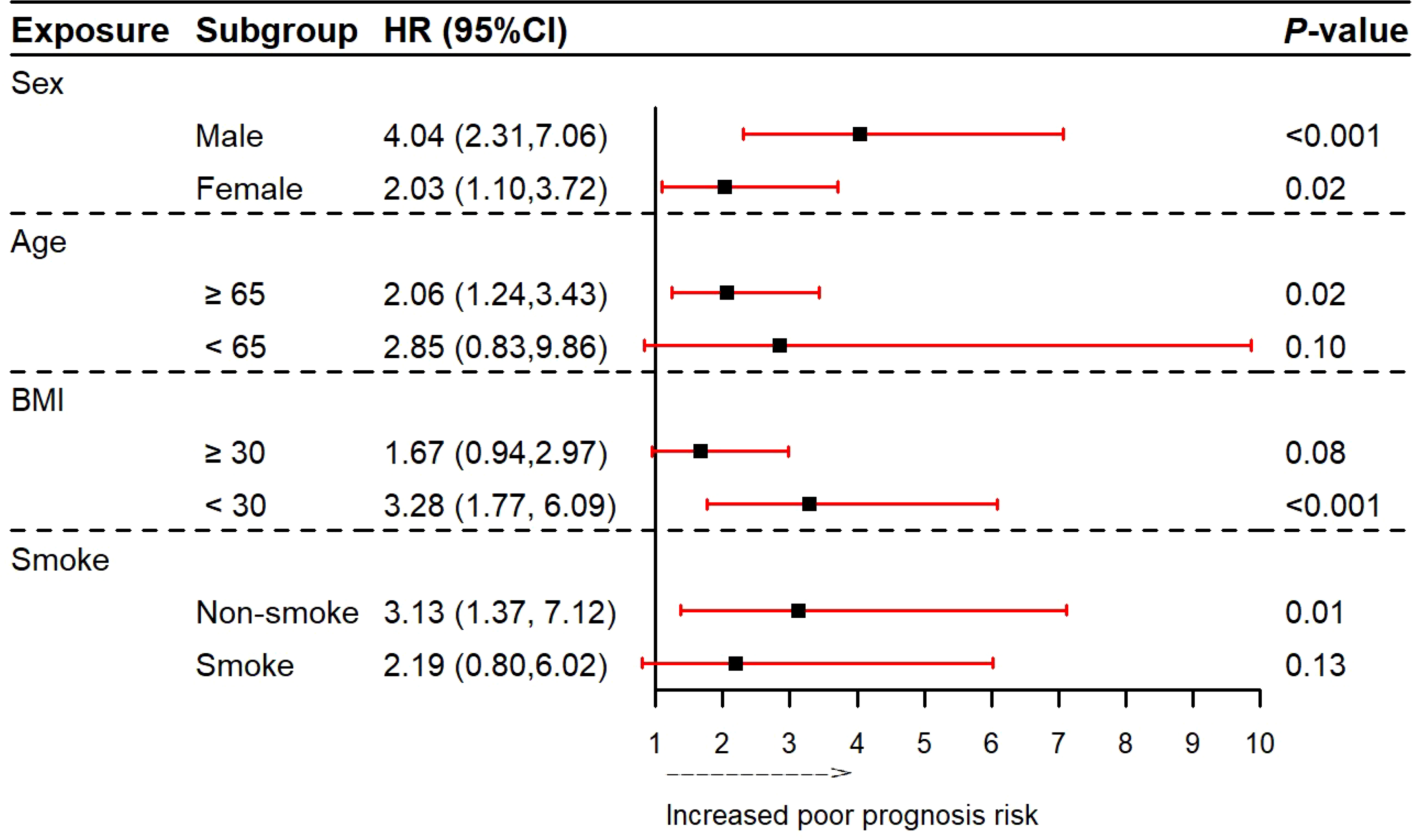
Forest plot of all-cause mortality according to different subgroups in DM people. FAR, fibrinogen-to-albumin ratio.

The median follow-up period for this study was 15.6 years. Restricted cubic spline suggested that the relationship between FAR and poor prognosis was linear in all patients (*P*-value overall = 0.000; *P*-value for nonlinearity = 0.111) and non-DM populations(*P*-value overall = 0.000; *P*-value for nonlinearity = 0.056), but was non-linear in the DM population (*P*-value overall < 0.001; *P*-value for nonlinearity = 0.016). In all ASCVD patients, the optimal FAR threshold was 0.92 derived from the restricted cubic spline function analysis. The prognosis of patients worsened with increasing FAR, particularly in the DM population (**Figure 4**). The subgroup analysis of DM patients showed that aathe FAR_H group of men or non-obesity patients had higher all-cause mortality rates (**Figure 5**).

## Discussion

In this retrospective observational study of the NHANES cohort from 1999–2002, we examined the relationship between ASCVD and poor prognosis in patients with different circulating fibrinogen-to-albumin ratio (FAR) levels. Patients with ASCVD who had higher FAR levels generally had worse outcomes in terms of long-term prognosis, especially in patients with ASCVD combined with DM. The survival rate of patients with ASCVD combined with DM who had higher FAR levels was significantly lower than the other three groups. This results strongly suggested that diabetes has an impact on the determination of a high or low FAR for long-term prognosis, particularly for the FAR_H group. Diabetes can synergize with the inflammatory response to produce a cascade amplification effect, which has an exacerbating effect on plaque progression and instability. In contrast, when FAR levels were low, ASCVD patients had a better prognosis than patients with high levels of FAR, and the presence or absence of comorbid DM had a lesser impact on all-cause mortality in ASCVD. In addition, the poor prognosis in the FAR_H/DM group was related to gender and BMI. Our findings suggest that ASCVD patients should be classified according to FAR levels to facilitate a more accurate predictive assessment.

Both circulating fibrinogen and albumin are reactants in the acute phase of inflammation. When inflammation occurs, fibrinogen levels rise dramatically, causing red blood cell aggregation and increased blood viscosity. This is associated with local activation of endothelial cells, accumulation of platelets, and recruitment of monocytes in the blood, which ultimately leads to vessel wall damage, further promoting inflammation and increasing the risk of atherosclerosis (14). Fibrinogen is one of the independent risk factors for cardiovascular disease. Cardiovascular diseases (CVD), such as coronary heart disease, heart failure, stroke, and hypertension, are associated with the formation of a dense fibrin network, which resists fibrinolysis. Dense fibrin networks are typical of acute patients during myocardial infarction or ischaemic stroke episodes. In contrast, hypofibrinolysis is a persistent fibrin feature in patients suffering from stable coronary artery disease (18, 19). Elevated fibrinogen levels lead to an increased risk of CVD and all-cause mortality (20), especially when combined with diabetes or in the early stages of diabetes (21). A prospective community-based cohort study in an Asian population suggested that fibrinogen may be a potential risk factor for coronary artery disease (CAD) (22). Marlien et al. demonstrated that fibrinogen was not only correlated with CVD in the cross-section but also strongly associated with all-cause mortality at follow-up (23). A study by Junxiu et al. also showed that higher levels of fibrinogen were associated with lower survival in patients with CVD (24). Albumin, synthesized by the human liver, maintains the stability of plasma colloid osmotic pressure and is a carrier for transporting many insoluble small organic molecules in the blood. Albumin is an acute-phase reactive protein that exhibits antioxidant and antiplatelet action *in vitro*. It also plays a crucial role in extracellular antioxidant defense against cardiovascular disease and hypertension. Low albumin levels are also thought to be an independent predictor of thrombotic burden in people with acute coronary syndrome (25). The potential impact of hypoalbuminemia in CVD mainly involves anti-inflammatory, antioxidant, anticoagulant, and anti-aggregation capacities (26). A study of the 1999-2018 NHANES database COPD population found that high albumin was associated with reduced all-cause mortality and mortality from CVD in patients (27). A meta-analysis revealed a strong independent connection between low plasma albumin and CVD, which could be explained by plasma albumin’s role as a negative acute phase reactant (28).

Fibrinogen and albumin are both present in the blood. Compared to other indicators of inflammation, the ratio of the two allows changes in both indicators to be observed simultaneously. It presents a more intuitive picture of the specifics of the relationship between fibrinogen and albumin in the blood. Because fibrin and albumin correlate positively and negatively, respectively, with the inflammatory response, their ratios are more sensitive and have a stronger correlation with the inflammatory response than other indicators. What’s more, FAR responds to the intravascular coagulation-fibrinolysis cascade and coagulation status, and may be able to better predict the onset of intravascular inflammation and coagulation (15, 29). FAR has been shown to predict poor prognosis in patients with gestational diabetes mellitus, ankylosing spondylitis, acute decompensated heart failure, atherosclerotic stroke of the large arteries, as well as other coronary artery diseases, breast cancer, and pancreatic cancer (13, 30–35). Mahmut et al. found that FAR was also a strong predictor of elevated blood pressure in patients with hypertension (14). FAR was an independent predictor of slow coronary blood flow (36). Fibrinogen-to-albumin ratio (FAR) is significantly associated with the severity and prognosis of cardiovascular disease (CVD) (37).

FAR was positively correlated with HbA1c, FPG, and other glucose metabolism indicators. High levels of FAR were associated with impaired glucose tolerance (5). Inflammation is a prevalent cause of CVD and DM, and chronic inflammation such as DM raises fibrinogen levels, increasing the likelihood of unfavorable outcomes in CAD patients (30). It has been demonstrated that FAR levels in male type-2 diabetes patients are independently and strongly linked with arterial stiffness, an early lesion of CVD. This is consistent with the current study’s findings that ASCVD patients in the FAR_H/DM group had increased all-cause mortality (37). In addition, research by Peizhi et al. on CAD patients having PCI indicated that the FAR-H/DM group had a greater risk of all-cause mortality and cardiac death than the other three groups (5). In our study, the all-cause mortality rate of ASCVD patients in the FAR_H/DM group was considerably greater than the other three groups, with an interaction between DM and FAR. Subgroup analysis of diabetic patients reveals which subgroup FAR is a better predictor of DM patients. We discovered that among men with a BMI of less than 30, the outcomes of ASCVD patients with varying levels of FAR changed significantly.

In ASCVD patients, especially those with DM, different levels of FAR are directly associated with the prognosis and survival of patients, and the outcome can be strongly predicted according to FAR level. Furthermore, anti-inflammatory therapy may be effective in improving the prognosis of ASCVD patients. As a result, the probable pathways by which FAR influences ASCVD prognosis, as well as focused FAR treatment, should be explored further.

This study has some limitations. First, FAR is a dynamically changing test, but the initial FAR used in this study does not reflect FAR changes during the follow-up period. At the same time, FAR is not a continuous dynamic variable, which makes it difficult to detect the development of inflammation at the individual level through changes in FAR. Second, this retrospective study was conducted among Americans, so the ethnic disparities might affect FAR’s predictive utility in different populations. And more research is needed to see if the findings can be generalized to other populations. Third, the exclusion of some individuals due to limitations of the database and inclusion-exclusion criteria resulted in unavoidable data bias. Fourth, the study population had a limited number of cases, which limits statistical power for granular analyses, and the small number of cases in each group after grouping based on FAR and DM could not be analyzed by further grouping based on gender and age. Fifth, we only examined the statistical relationship between FAR and mortality, and the underlying biological mechanisms requires further experimental studies.

## Conclusions

Higher level of FAR was independently associated with 15-year mortality among ASCVD patients with DM. The combination of FAR in patients with ASCVD and DM may assist in risk stratification and prognostic assessment.

## Data Availability

The original contributions presented in the study are included in the article/**Supplementary Material**. Further inquiries can be directed to the corresponding author.
