# Supplementary material for: Fibrinogen-to-albumin ratio predicts mortality in patients with diabetes mellitus and atherosclerotic cardiovascular disease
Source: Front Endocrinol (Lausanne). 2025 May 29;16:1539114. doi: 10.3389/fendo.2025.1539114 (PMC12158729; doi:10.3389/fendo.2025.1539114)
Supplement: Supplementary file 1 [file Table1.docx]

Table S1. Baseline characteristics of study participants according to FAR and DM

| Characteristics | Total | FAR_L/non-DM | FAR_L/DM | FAR_H/non-DM | FAR_H/DM | *P*-value |
| --- | --- | --- | --- | --- | --- | --- |
| Age, years | 66.132 (0.792) | 63.974 (1.048) | 64.571 (1.432) | 68.065 (1.331) | 69.667 (1.434) | 0.013 |
| BMI, kg/m2 | 29.301 (0.302) | 27.678 (0.293) | 30.776 (0.800) | 29.539 (0.587) | 32.633 (0.877) | < 0.001 |
| SBP, mmHg | 133.942 (1.123) | 132.043 (1.798) | 132.597 (2.324) | 135.392 (1.443) | 137.594 (2.779) | 0.179 |
| DBP, mmHg | 69.922 (0.777) | 71.268 (1.132) | 69.150 (1.643) | 70.471 (1.031) | 65.365 (2.160) | 0.09 |
| HbA1c, % | 5.957 (0.038) | 5.437 (0.019) | 7.212 (0.206) | 5.492 (0.027) | 7.471 (0.168) | < 0.001 |
| fasting blood glucose, mmol/L | 6.038 (0.116) | 5.247 (0.063) | 7.965 (0.548) | 5.159 (0.057) | 8.687 (0.437) | < 0.001 |
| low-density lipoprotein cholesterol, mmol/L | 3.041 (0.077) | 2.974 (0.090) | 2.758 (0.204) | 3.264 (0.143) | 2.909 (0.165) | 0.147 |
| TG, mmol/L | 1.988 (0.078) | 1.861 (0.094) | 3.107 (0.541) | 1.706 (0.090) | 2.177 (0.138) | 0.015 |
| Gender |  |  |  |  |  | < 0.001 |
| Female, n (%) | 337 (41.048) | 108 (42.116) | 31 (28.390) | 120 (52.633) | 78 (58.165) |  |
| Male, n (%) | 484 (58.952) | 195 (57.884) | 76 (71.610) | 136 (47.367) | 77 (41.835) |  |
| Race |  |  |  |  |  | < 0.001 |
| Black, n (%) | 143 (17.418) | 32 (5.162) | 13 (6.344) | 46 (9.495) | 52 (22.219) |  |
| Mexican American, n (%) | 134 (16.322) | 45 (2.340) | 27 (4.000) | 30 (1.804) | 32 (5.439) |  |
| Other, n (%) | 43 (5.238) | 15 (5.776) | 6 (6.221) | 15 (7.805) | 7 (5.698) |  |
| White, n (%) | 501 (61.023) | 211 (86.722) | 61 (83.434) | 165 (80.897) | 64 (66.643) |  |
| Alcohol.user, n (%) |  |  |  |  |  | 0.117 |
| 0 | 128 (16.410) | 35 (12.559) | 15 (17.924) | 44 (17.457) | 34 (23.400) |  |
| 1 | 652 (83.590) | 256 (87.441) | 87 (82.076) | 199 (82.543) | 110 (76.600) |  |
| Hypertension, n (%) |  |  |  |  |  | 0.013 |
| 0 | 145 (17.661) | 66 (26.479) | 15 (13.828) | 51 (22.513) | 13 (9.036) |  |
| 1 | 676 (82.339) | 237 (73.521) | 92 (86.172) | 205 (77.487) | 142 (90.964) |  |
| Edu, n (%) |  |  |  |  |  | < 0.001 |
| less than 12 years | 352 (42.979) | 106 (27.771) | 57 (38.497) | 104 (33.298) | 85 (55.048) |  |
| 12 years | 286 (34.921) | 121 (47.120) | 23 (26.860) | 103 (46.095) | 39 (26.798) |  |
| more than 12 years | 181 (22.100) | 76 (25.109) | 27 (34.643) | 48 (20.606) | 30 (18.155) |  |
| FamilyCVD, n (%) |  |  |  |  |  | 0.881 |
| 0 | 675 (82.217) | 245 (77.701) | 91 (81.080) | 209 (77.924) | 130 (81.435) |  |
| 1 | 146 (17.783) | 58 (22.299) | 16 (18.920) | 47 (22.076) | 25 (18.565) |  |
| FamilyDM, n (%) |  |  |  |  |  | < 0.001 |
| 0 | 405 (49.330) | 173 (56.067) | 33 (27.713) | 145 (52.866) | 54 (33.109) |  |
| 1 | 416 (50.670) | 130 (43.933) | 74 (72.287) | 111 (47.134) | 101 (66.891) |  |
| DM, n (%) |  |  |  |  |  | < 0.001 |
| 0 | 559 (68.088) | 303 (100.000) | 0 (0.000) | 256 (100.000) | 0 (0.000) |  |
| 1 | 262 (31.912) | 0 (0.000) | 107 (100.000) | 0 (0.000) | 155 (100.000) |  |
| Smoke, n (%) |  |  |  |  |  | 0.254 |
| non-smoking | 330 (40.195) | 104 (34.552) | 51 (42.783) | 104 (39.273) | 71 (48.339) |  |
| smoking | 491 (59.805) | 199 (65.448) | 56 (57.217) | 152 (60.727) | 84 (51.661) |  |
| Hyperlipidemia, n (%) | |  |  |  |  | 0.203 |
| 0 | 106 (12.911) | 43 (11.378) | 10 (4.642) | 37 (13.690) | 16 (8.940) |  |
| 1 | 715 (87.089) | 260 (88.622) | 97 (95.358) | 219 (86.310) | 139 (91.060) |  |
| Cancer, n (%) |  |  |  |  |  | 0.448 |
| 0 | 664 (80.877) | 248 (82.983) | 86 (78.038) | 200 (78.029) | 130 (84.269) |  |
| 1 | 157 (19.123) | 55 (17.017) | 21 (21.962) | 56 (21.971) | 25 (15.731) |  |

Abbreviation: FAR, fibrinogen-to-albumin ratio; BMI, body mass index; SBP, systolic blood pressure; DBP, diastolic blood pressure; HbA1c, glycated hemoglobin; CVD, cardiovascular disease; DM, diabetes mellitus
